# A MATLAB Algorithm to Automatically Estimate the QT Interval and Other ECG Parameters and Validation Using a Machine Learning Approach in Congenital Long-QT Syndrome

**DOI:** 10.1007/s12265-025-10693-0

**Published:** 2025-09-30

**Authors:** Elinor Tzvi-Minker, Sven Dittmann, Corinna Rickert, Andreas Keck, Eric Schulze-Bahr

**Affiliations:** 1 Syte Institute, Hamburg, Germany; 2 Department of Cardiovascular Medicine, Institute for Genetics of Heart Diseases (IfGH), University Hospital Münster, Münster, Germany

**Keywords:** Long-QT syndrome (LQTS), Electrocardiogram (ECG), MATLAB algorithm, Machine learning, T-wave

## Abstract

Myocardial repolarization and QT duration are crucial markers for diagnosis and monitoring of congenital long QT syndrome (LQTS). Here we present a novel algorithm to automatically estimate the QT interval based on Lepeschkin’s tangent method, as well as parameters underlying T-wave morphology in digital electrocardiograms (ECGs) of 466 patients with LQTS. The algorithm’s performance was validated using ECG data from 40 healthy controls. The results were compared against expert measurement of the QT interval, as well as against the results of the ECG device MUSE™ system. Applying an optimizable Support Vector Machine classifier on the algorithm’s outcomes achieved an accuracy of 78.1% and area under the curve of 0.85 in classifying LQTS patients with a prolonged QTc interval (upon QT_GS) from those with a normal QTc interval. The presented MATLAB®-based algorithm offers a transparent and reproducible approach to automatic QT interval estimation and QTc calculation in LQTS patients, potentially improving automatized screening, diagnostic precision and patient management.

**Graphical Abstract:**

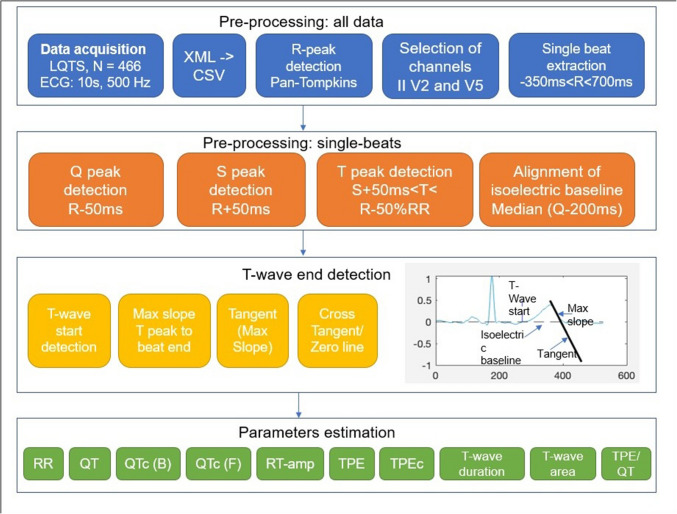

**Supplementary Information:**

The online version contains supplementary material available at 10.1007/s12265-025-10693-0.

## Introduction

Long-QT Syndrome (LQTS) is a genetically heterogeneous, inherited cardiac arrhythmia syndrome, typically characterized by prolongation of the QTc interval on the surface electrocardiogram (ECG). In addition, T-wave abnormalities, and a propensity to develop polymorphic ventricular tachyarrhythmias are common. These may cause triggered syncope, sudden cardiac arrest, or death. In addition to congenital forms, also acquired conditions are known to cause secondary and potentially clinically relevant forms of disturbance in myocardial repolarization. From a clinical point-of-view, early detection of LQTS or abnormal myocardial repolarization is crucial to implement a tailored treatment, prevent life-threatening arrhythmias, detect genetic subforms and offer genetic counseling. The latter can reduce the risk of sudden cardiac death (SCD) in affected individuals and related, potentially affected, family members. Correct and precise detection of LQTS in the surface ECG can be, however, challenging and resemble a random finding, e.g., when associated with non-specific symptoms, in the setting of intermittent, heart-rate dependent ECG changes, including so-called concealed QTc prolongation [4, 15], and due to a genetic complexity [8]. Furthermore, the lack of routine ECG screening [16] and reliance on manual measurements or automatic estimations by ECG analysis software, leads to unrecognized or misjudged diagnoses of LQT syndrome. Of note, a correct determination of the QT interval is not homogeneous, even by clinical experts [9].

The QT interval is defined between the onset of the Q-wave and the offset of the T-wave in the surface ECG, measured in a baseline 12-lead or exercise ECG. The Bazett’s formula is commonly used to correct the measured QT interval by the heart-rate (HR) (so-called QTc interval duration), and is still preferred over other corrections methods for LQT1 and LQT2 [3], two genetic subtypes that are each causative for 30–35% of all LQTS cases [1]. At higher HR, the Bazett’s formula tends to result in longer QTc values, whereas at lower HR, QTc values are shorter when compared to other methods of QT interval correction.

The T-wave end, which marks the end of ventricular repolarization, is a critical component in determining the QT interval. Thus, accurate and automated detection of the T-wave end is imperative for correct diagnosis and effective monitoring and management of LQTS patients. Traditionally, the determination of the T-wave end has been reliant on manual measurements, which are time-consuming and susceptible to intra- and inter-observer variability. Indeed, most physicians, including cardiologists, have difficulties to correctly identify a prolonged QT interval [12], probably due to the complexity and variability of T-wave morphologies in LQTS patients and genetic subforms. To date, different methods have been used to correctly assess the T-wave end [11, 17]. Importantly, Lepeschkin’s ‘tangent method’ has been shown to be unaffected by the electrical heart axis orientation and applied on a beat-to-beat basis regardless of the T-wave morphology. It is therefore the preferred method for T-wave end determination by numerous electrophysiologists [17].

Recently, several automated algorithms have been developed to detect the T-wave end, based on a variety of measures [6]. Of note, current commercial ECG management systems measure the QT interval by automated algorithms based on the average of a median complex over a period of time [10]. Consequently, beat-to-beat detection algorithms which include the QT-interval dynamicity have been published, but often use only a single ECG lead (mostly II or V5), which makes the QT interval susceptible to heart axis orientation and electrode placement.

Here, we developed a MATLAB® (2021a, The MathWorks, Natick, MA, USA)-based algorithm to specifically and automatically detect the end of the T-wave in three ECG channels (ECG leads II, V2 and V5) as a marker of repolarization in single beats using the ‘tangent method’. Thereby, the MATLAB® algorithm automatically determines various ECG parameters such as the QT interval, HR for subsequent calculation of the corrected QT interval corrected according either to Bazett (QTc (B)) or to the Fridericia (QTc (F)) formula, T-peak to T-end, the T-wave duration, and the T-wave area. In addition, the R-peak to T-peak amplitude ratio is also calculated.

These unique T-wave parameters reflect myocardial repolarization and may additionally contribute to the clinical expression of LQTS. Notably these parameters are usually not estimated in commercial systems. We then investigate the contribution of these parameters to a correct evaluation of LQTS using machine-learning methods.

## Methods

### Clinical Data and ECG Recordings

In this study, anonymized 12-lead ECG recordings in supine position from 466 LQTS patients and 40 healthy control subjects were included. Patients had a confirmed pathogenic or a likely pathogenic variant either in the *KCNQ1* gene (genetic LQT1 subtype, N = 318), in the *KCNH2* gene (genetic LQT2 subtype, N = 141) or in the SCN5a gene (genetic LQT3 subtype, N = 6). Data were collected during routine outpatient care at the Institute for Genetics of Heart Diseases of the University Hospital Münster, Germany. The study was conducted in accordance with the Declaration of Helsinki (as revised in 2013) and approved by institutional ethics board of University Hospital Münster, Münster, Germany (2021–315-f-S). An informed consent was signed by all study participants. ECG datasets were excluded from further analysis when data quality was low (assessed manually by study investigators). A manual measurement of the QT interval duration was performed by experienced team members on a standard rhythm strip (speed: 50 mm/s), focusing on ECG leads II and V5. In case the T-wave end was not clear enough to separate, lead V2 was chosen for assessment of the QT. This manual measurement served as a Gold Standard (GS) for evaluation of algorithm results.

### Data Acquisition and Pre-Processing

**Fig. 1:**
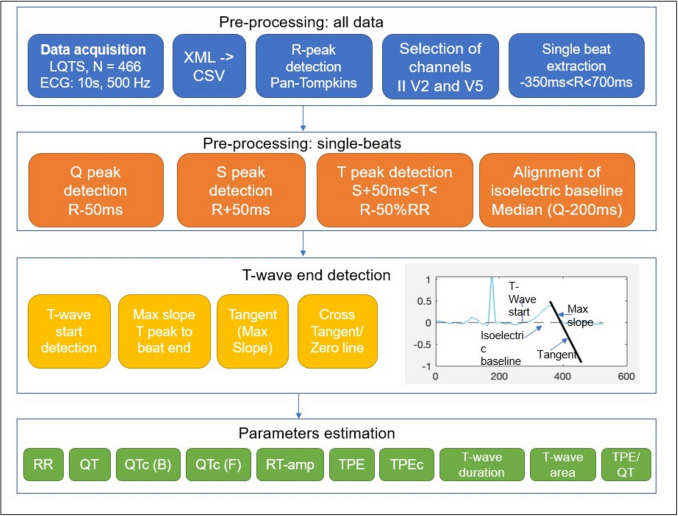
Overview of the pre-processing and analysis steps for automated detection of the t-wave end and evaluation of ECG parameters

Pre-processing steps and subsequent analyses of the raw ECG data are shown in Fig. 1. 12-lead ECGs were recorded for 10 s with a 500 Hz sampling frequency using an ECG machine (GE CAM-HD Registrator, GE Cardiosoft V6.73, GE HealthCare). The data was stored using the MUSE™ data management system (Muse v9 Cardiology Information System, GE HealthCare) and later exported in an anonymized form in XML format. Subsequently, XML files were converted into CSV files using freely available Python script.^1^

### Description of the Algorithm

**Fig. 2:**
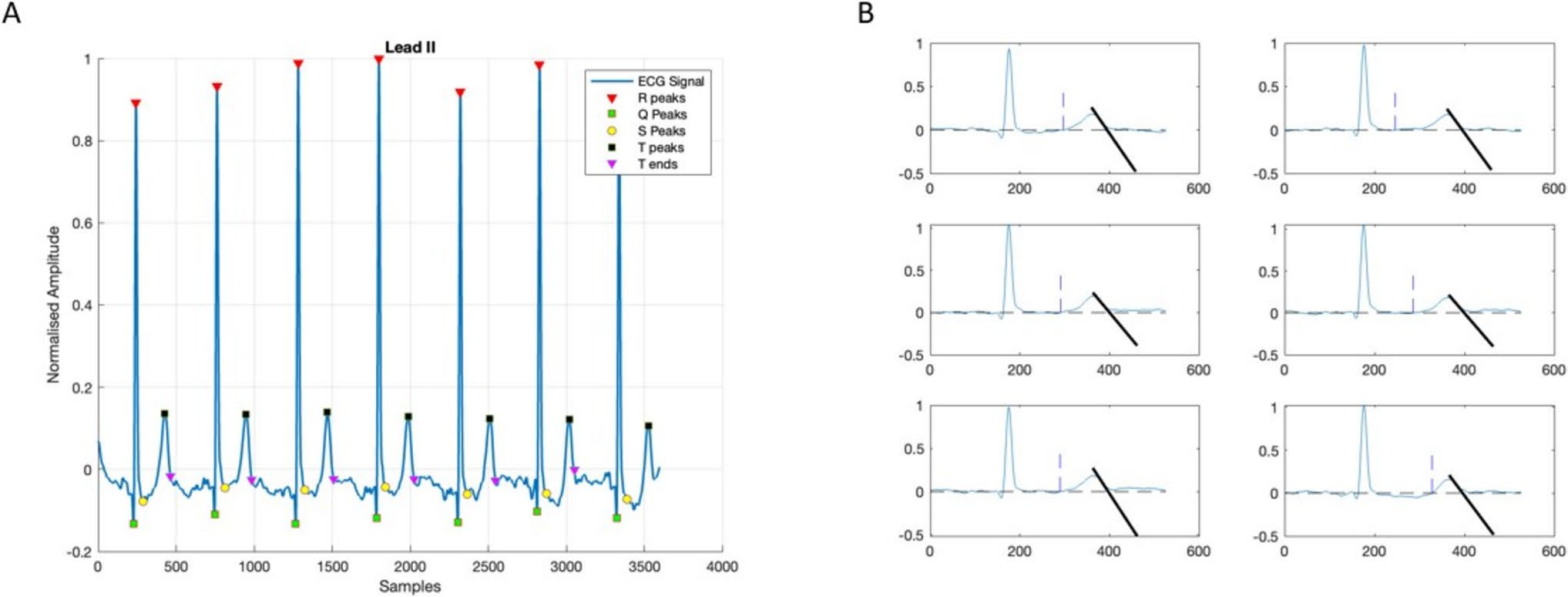
Plots produced by the MATLAB® algorithm in an exemplary patient. Plot A shows the normalized ECG from Lead II with the respective annotations for QRS peak locations (green, red and yellow resp.) as well as T start and T end locations (black and purple resp.). Plot B shows the beat-by-beat detection of the T-wave end according to the tangent method. The tangent is annotated as a black line. The baseline is annotated with a dashed line

Next, a method to detect accurately the T-wave end was developed using custom-made MATLAB® scripts and open-source routines. The output is shown in Fig. 2 from two different LQTS patients.

The following pre-processing steps were taken:

1. A lowpass filter and a notch filter were applied to remove both high-frequency noise as well as power line noise (f = 50 Hz)
2. R-peaks were identified using a MATLAB® implementation of Pan-Tompkins algorithm.^2^
3. Beats were windowed according to the R-peak locations with 350 ms before R-peak until 700 ms after the R-peak.
4. Isoelectric baseline of single beats was aligned with the zero line by subtracting the median of the signal amplitude in a window of 200 ms before the Q-peak. Usually, the TP segment is chosen for the isoelectric baseline, but since we cannot always reliably detect the P-wave, the Q-peak was selected. To account for the cases that a P-wave does appear, a larger time-window and the median were selected to calculate the isoelectric baseline. In Fig. 2C-D, the isoelectric baseline can be observed in an exemplary patient with 6 extracted beats.

Next, the QRS complex as well as other ECG markers were searched for three lead channels II, V2 and V5:

1. Q- and S-peaks were defined as the local minimum within a 50 ms window before and after the R-peak respectively.
2. T-peak was searched within a delay of 50 ms following S-peak and 50% of the RR distance (in sec) before the next R-peak. We accounted for both, negative and positive peaks. In addition, we defined that a T-peak should be at least 10% of the amplitude of the preceding R-peak. This step helps to distinguish the T-peak from noise.
3. For detection of the T-wave start and T-wave end, we followed these steps:
  1. T-wave start was defined to be the first point within a search window between S-wave peak and T-wave peak that crossed the isoelectric line (adjusted to whether the T-wave peak is positive or negative).
  2. The steepest point of the descending or ascending limb of the T-wave (depending on whether the peak is positive or negative) was searched using the differential of the amplitude within a time window between the T-wave peak and the end of the beat (defined above).
  3. The tangent through this point was calculated and the cross point between this line and the zero baseline was searched.
  4. The first sample of the signal following this point was marked as the T-wave end.

### Calculation of Further ECG Parameters

We then calculated the following parameters:

1. T-wave area was calculated using an approximation of the integral of the signal from T-wave start to end using the trapezoidal method (spacing: 2 ms).
2. T-peak to T-end duration (TPE) was defined as the time between the T-wave peak and the T-wave end. The TPE was then HR-corrected as follows:
  $TPEc=\frac{TPE}{\sqrt{RR}}$, with RR interval is 60,000/HR, calculated in ms. We removed all data points below TPEc = 40 ms.
3. QT interval was defined as the duration between the beginning of the Q-wave peak (if absent: of the R-wave) and the T-wave end.
4. We then calculated the median of these values across all interval beats. The QTc interval was HR-corrected according to Bazett formula (QTc(B)): $QTc=QT/\sqrt{RR}$ as well as according to the Fridericia formula (QTc(F)): $QTc=QT/\sqrt[3]{RR}.$
  The RR interval is 60/HR, calculated in seconds.
5. Next, we calculated the amplitude ratio between the R-peak and the T-peak.
6. Finally, the ratio TPE/QT (uncorrected) was calculated.

### Validation of the Algorithm

**Fig. 3:**
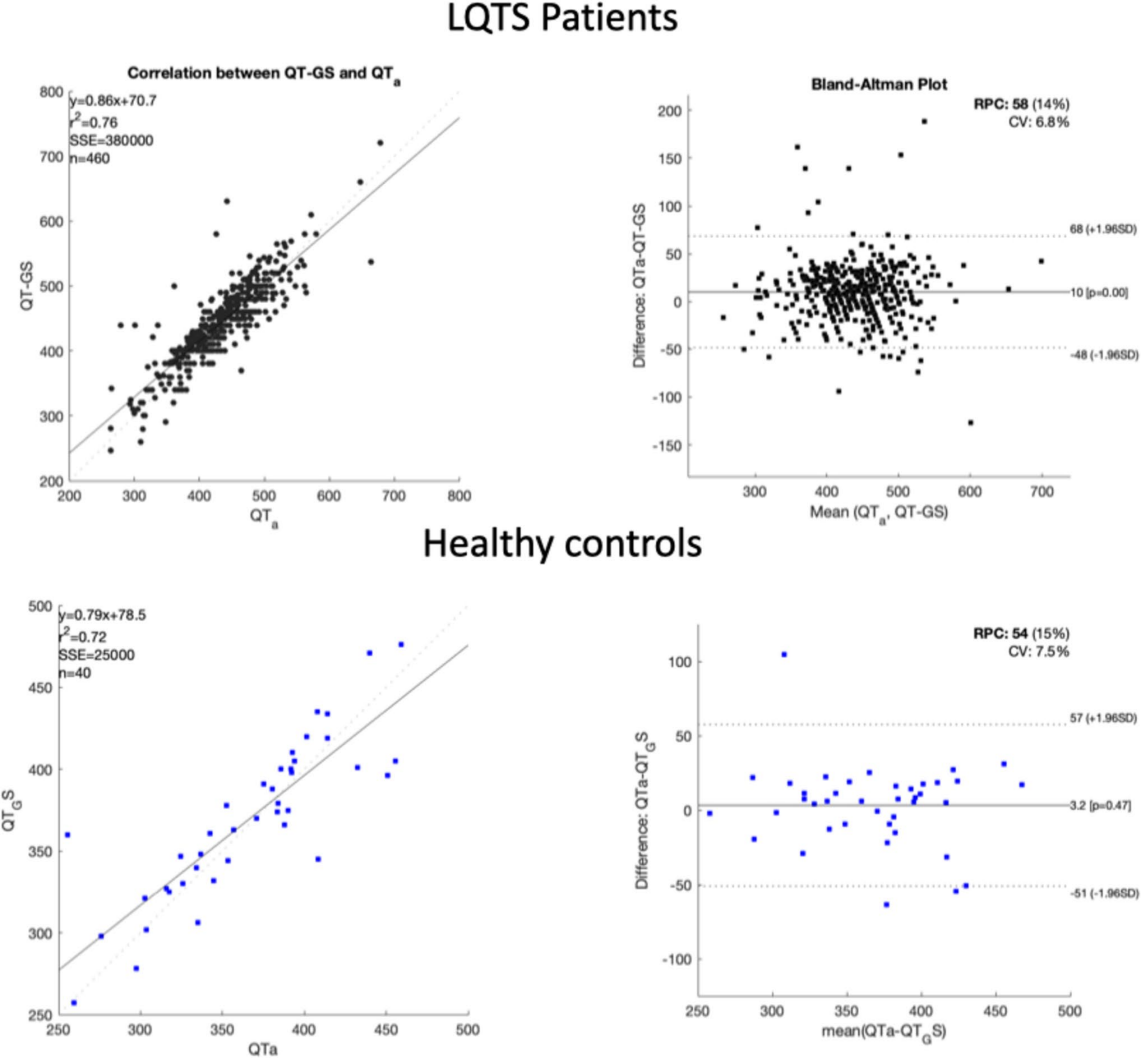
Bland–Altman and correlation plot for the comparison between algorithm estimation of the QT and Gold Standard QT

**Fig. 4:**
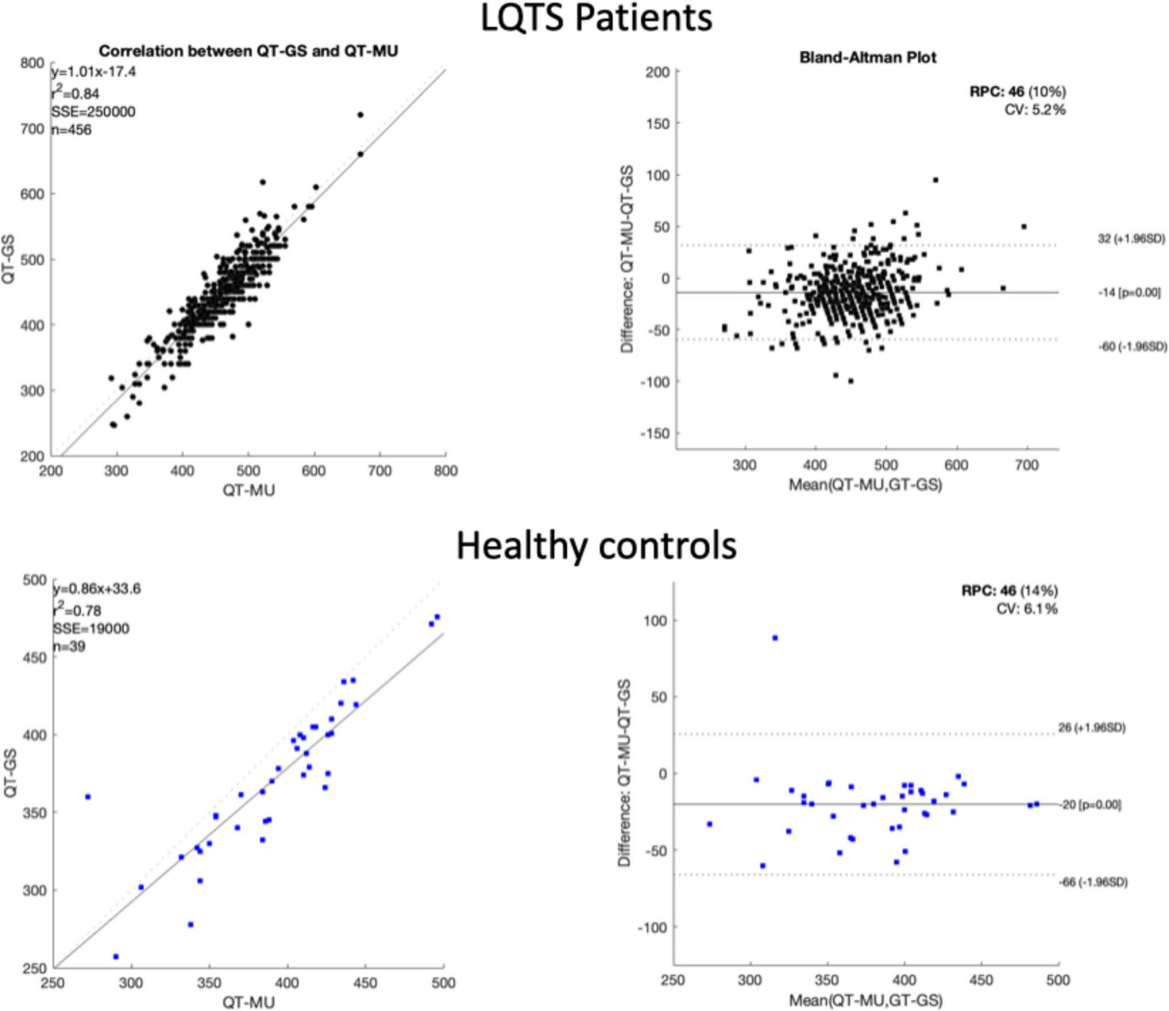
Bland-Altman and correlation plot for the comparison between MUSE (TM) system estimation of the QT and Gold Standard QT

To assess the validity of our algorithm in correctly calculating the QT interval, we extracted both the QT and QTc (corrected using the Fridericia method, i.e., QTc(F)) from the XML files of each patient, as assessed by the MUSE™ system. In addition, a manual measurement of the QT interval duration was independently performed by expert cardiologists to serve as a Gold Standard (GS) for comparisons with the algorithm output. QT was obtained from one of three ECG channels II, V2 and V5, according to the following logic: if the algorithm could assess the QT on all channels, then the value of channel II was chosen. Otherwise, if no value was determined for II (due to noise or a lack of a T-wave on that channel), V5 was chosen. If on both channels, no value could be determined, the value of QT on V2 was chosen. Next, we used a Bland–Altman plot (Fig. 3) to assess the differences in each patient between the QT as assessed by our algorithm (QT_a_) in channels II, V2 and V5 and QT assessed by the experts (QT_GS). Similarly, a Bland–Altman plot (Fig. 4) was used to assess the difference between QT as assessed by MUSE™ system (QT_MU) and QT_GS. The Bland–Altman plot helps to visualize any systematic bias between the two methods by plotting the differences against the averages of the two measurements. This allows for easy identification of any consistent over- or underestimation by one method compared to the other.

Next, we assessed the probability of the algorithm to correctly detect prolonged QTc interval durations as diagnostic feature of LQTS using supervised machine-learning. To this end, we evaluated the algorithm outputs including QT but also other parameters indicating T-wave morphology (see Fig. 1. Parameters estimation). The genotype positive LQT1 and LQT2 patients were tagged according to their gender (female, male) and GS_QT values into two groups: those with a normal QTc(B), i.e., females: < 460 ms, males: < 450 ms, or prolonged QTc, i.e., females: > 460 ms, males > 450 ms.

As features we used the above mentioned 10 parameters: TPE, TPEc(B), TPE/QT, QT, QTc(B), QTc(F), RR interval, R-peak to T-peak amplitude ratio, T-wave area, and T-wave duration from the selected ECG channel (see above). We then used the classifier learner toolbox by MATLAB® to investigate to which degree the ECG parameters can correctly distinguish LQT1 patients with normal QTc from those with a prolonged QTc as determined by the QTc_GS.

Subsequently, we tested this hypothesis with the following machine-learning algorithms: Support Vector Machine (SVM), k-nearest neighbors (KNN), Linear Discriminant Analysis (LDA), Decision Tree (DT), Logistic Regression (LR), Naive Bayes (NB) and Ensemble. To this end, the data were first split into training and validation sets. The training set was used to fit the model, learning the parameters that best describe the relationship between predictors and the response variable. As models are trained, various performance metrics are estimated such as model accuracy and the area under the curve. These metrics assess the classification accuracy. Next, the dataset was divided into 5 equally sized folds for the cross-validation. For each fold, the model is trained on 4 folds and validated on the remaining fold. This process is repeated 5 times, with each fold being used exactly once as the validation data.

## Results

Manual assessment of T-wave end and subsequently measurement of the QT interval was performed in all LQTS patients and healthy controls. In Tables 1 and 2 we present the manually measured QT interval as well as correction according to Bazett (B) and Fridericia (F) in the analyzed cohorts, separated by gender and by the QTc length.

**Table 1:** Demographics, manual and MUSE™ system assessment of QTc values in LQT1 patients

	N	QTc (B) GS Mean ± SD (ms)	QTc (F) GS Mean ± SD (ms)	QTc (F) MU Mean ± SD (ms)	QT MU Mean ± SD (ms)	QT GS Mean ± SD (ms)
N-analyzed	466	466	466	456	456	466
Female	302 (64.8%)	458.9 ± 36.1	455.6 ± 37.7	471.5 ± 28.0	463.9 ± 48.9	450.3 ± 55.5
Male	164 (35.2%)	444.3 ± 37.5	440.6 ± 40.2	457.7 ± 29.3	450.3 ± 57.4	435.6 ± 62.8
LQTS patients with a normal QTc (B)	252 Female: 161 (63.9%) Male: 91 (36.1%)	426.9 ± 23.2	425.8 ± 27.1	449.6 ± 23.5	447.5 ± 50.0	425.2 ± 51.9
LQTS patients with a prolonged QTc (B)	214 Female: 141 (65.9%) Male: 73 (34.1%)	483.8 ± 25.1	477.8 ± 31.7	486.9 ± 21.3	472.9 ± 51.9	467.4 ± 57.5

*N* Number of patients, *SD* Standard deviation, *QT* *MU* Assessment of QT by the MUSE™ system, uncorrected. *QT GS* Gold standard of QT manual measurement by experts, uncorrected. QTc (B) *GS* Gold standard of QT manual measurement by experts, corrected using Bazett, QTc (F) *GS* Gold standard of QT manual measurement by experts, corrected using Fridericia. QTc MU – assessment of QT by the MUSE™ system, corrected using Fridericia

**Table 2:** Demographics, manual and MUSE™ system assessment of QTc values in healthy controls

	N	QTc (B) GS Mean ± SD (ms)	QTc (F) GS Mean ± SD (ms)	QTc (F) MU Mean ± SD (ms)	QT MU Mean ± SD (ms)	QT GS Mean ± SD (ms)
N-analyzed	40	40	40	39	39	40
Female	24 (60%)	406.0 ± 25.0	397.4 ± 36.8	415.2 ± 28.0	384.3 ± 48.7	363.8 ± 45.8
Male	16 (40%)	395.7 ± 28.9	387.7 ± 32.2	410.6 ± 23.0	401.5 ± 48.9	377.7 ± 52.7

*QT MU* – Assessment of QT by the MUSE™ system, uncorrected.* QT GS* – Gold standard of QT manual measurement by experts, uncorrected

*Note that per definition the healthy subjects have a QTcB smaller than the gender-specific threshold

### MATLAB® Algorithm Performance

The algorithm was able to determine the T-wave end and the other determined parameters in at least one of the three ECG channels across 460 out of 466 patients (98.7%) and in all 40 healthy subjects (100%). We excluded patients from further analyses in which the algorithm did not detect any T-wave (N = 6). One patient of the LQTS cohort was excluded due to a technical failure in the XML file. Five additional patients of the LQTS cohort were excluded due to data quality or a lack of a visible T-wave. Refer to the supplementary materials for further analysis of these cases and a detailed description.

Determining the final QT interval by the algorithm followed this logic: first, channel II was assessed. If no value was produced such as in N = 78 patients and N = 2 healthy subjects, channel V5 was assessed, if here too no value was produced and this was the case in N = 42 patients (in healthy subjects there was no such case), channel V2 determined the QT interval. The selection of different leads to determine the QT is a common practice and is validated through the strong correlation between the different leads: channel II and V5 (R = 0.97), channel V2 and V5 (R = 0.91) and channel II and V2 (R = 0.86).

In Table 3 we present the results of the QT estimation by the algorithm, as well as the estimation of additional T-wave morphology parameters, divided by gender and by cohort (LQTS patients and healthy controls).

**Table 3:** ECG parameters as assessed and calculated by MATLAB® algorithm (mean ± SD) in 460 LQT patients and 40 Healthy controls

Parameter	Female LQTS patients (N = 302)	Male LQTS patients (N = 164)	Female Healthy controls (N = 24)	Male Healthy controls (N = 16)
QT_a_ (ms)	440.7 ± 55.7	425.1 ± 62.9	357.1 ± 50.0	379.8 ± 53.3
RR interval (ms)	974.3 ± 200.2	968.2 ± 234.9	783.9 ± 184.0	947.6 ± 226.1
QT_a_c (B) (ms)	449.3 ± 39.0	435.8 ± 39.4	406.0 ± 24.4	393.4 ± 36.1
QT_a_c (F) (ms)	445.9 ± 40.1	431.4 ± 41.8	388.4 ± 29.3	388.2 ± 37.1
TPE (ms)	74.4 ± 18.7	73.7 ± 16.9	66.7 ± 8.7	74.0 ± 15.6
TPE to QT ratio	0.17 ± 0.04	0.17 ± 0.03	0.19 ± 0.02	0.19 ± 0.03
TPEc (B) (ms)	76.7 ± 19.4	76.4 ± 15.1	76.7 ± 10.4	77.2 ± 16.1
T-wave duration (ms)	250.3 ± 72.5	274.3 ± 77.4	212.7 ± 53.6	261.1 ± 76.4
T-wave area (no unit)	12.5 ± 11.8	13.5 ± 13.4	9.1 ± 6.7	10.6 ± 5.4
R to T amplitude ratio	4.4 ± 3.1	4.8 ± 5.0	3.9 ± 2.9	1.7 ± 2.4

QT_a_ – QT interval without correction, QT_a_c (B) – QT interval corrected according to Bazett, QT_a_c (F)—QT interval corrected according to Fridericia, TPE – T-peak to T-end interval, TPEc (B) – TPE corrected according to Bazett

To assess the performance of our algorithm in correctly estimating the QT interval, we calculated the individual differences between QT_a_ and QT_GS (uncorrected). This resulted in −10 ± 29.8 ms for the LQTS cohort and −3.2 ± 27.7 ms for the healthy controls (mean ± STD). Note that performance was significantly better for LQT1 patients with −5.5 ± 25.6 ms compared to LQT2 patients with −20.6 ± 35.2 ms. This means our algorithm estimates the QT interval shorter than the GS. Note however, that this value might be driven by outliers.

On the other hand, the difference between QT_MU and QT_GS for the LQTS cohort was: 13.8 ± 23.3 ms and for the healthy controls 20.2 ± 23.4 ms. In consequence, the QT_MU ECG software will generally produce a longer QT interval duration compared to QT_GS, which in principle means an over-diagnosis of individuals that do not have a long-QT syndrome.

Similarly, the difference between QT_a_C (F) and QTc_GS (uncorrected) for the LQTS cohort was: –8.8 ± 32.2 ms and for the healthy controls: −5.2 ± 30.9 ms. The difference between QTc (F)_MU and QTc (F)_GS for the LQTS cohort was: 16.6 ± 1.4 ms and for the healthy controls: 19.2 ± 28.4 ms. This suggests that correction based on HR leads to even better accuracy of our algorithm, whereas for the MUSE (TM) system, an even worse performance is observed.

The distribution of single-subject differences (both LQTS patients and healthy controls) to QT_GS are shown in the Bland–Altman plot in Fig. 3. Note that in very few cases (N = 7 in the LQTS cohort, and N = 1 in the healthy controls) the algorithm produced QT intervals that are more than 100 ms different than the GS. In those cases, the data quality was very low, prohibiting correct QT assessment. Nevertheless, the data were included in the assessment of the algorithm as the algorithm provided a value for the QT. Similarly, we show in Fig. 4 the Bland–Altman plot for QT_MU when compared to GS for both LQTS patients and healthy controls. We found a clear trend which may underlie systematic differences in lower QT intervals compared to higher QT intervals. No such systematic difference was observed in the results from our algorithm as shown in Fig. 3.

### Actual LQTS Detection by the Algorithm

From the 460 analyzed LQTS patients, N = 161 were identified as having a prolonged QT and N = 299 had a normal QT. We defined the following to calculate the algorithm’s performance:

- True positives (TP) are correctly identified patients with a prolonged QTc.
- True negatives (TN) are correctly identified individuals with a normal QTc.
- False positives (FP) are wrongly identified individuals with a prolonged QTc.
- False negatives (FN) are wrongly identified patients with a normal QTc.
- For our algorithm the respective performance was: FP = 27, FN = 82, TP = 134, TN = 217. The accuracy is therefore 76.3%, sensitivity is 62% and specificity is 89%.

According to the MUSE (TM) system, N = 293 were identified as having a prolonged QTc and N = 167 had a normal QTc, resulting in FP = 97, FN = 20, TP = 196, TN = 147. The accuracy is therefore lower with 74.6%, sensitivity is 90% and specificity is 60%. This means that our algorithm is in general more accurate and has a stronger specificity, with a very low false positive rate. However, it lacks in sensitivity, which means that some cases with a prolonged QTc interval will be identified as having a normal QTc interval.

### Validation of the Algorithm’s T-wave Parameters using Machine Learning

To account for T-wave parameters (listed above) as calculated by our algorithm, we used a machine learning approach. To this end, we used different machine learning methods to classify the T-wave parameters assessed by our algorithm to prolonged and normal QTc according to GS in the LQTS cohort. The best performing algorithm was optimizable Support Vector Machines (SVM) with a linear kernel. The classification had an accuracy of 78.1% and AUC of 0.85. This means that the T-wave parameters are valuable in assessing prolonged QT and that our algorithm is able, with a relatively high accuracy, to correctly estimate these parameters. Optimization of the hyperparameters was performed with 30 iterations. The results of all classification algorithms are presented in Table 4.

**Table 4:** Classification accuracy and area under the curve (AUC) results for each tested algorithm

	Accuracy	AUC
Optimizable decision tree (DT)	76.8%	0.76
Optimizable linear discriminant analysis (LDA)	77.7%	0.85
Logistic regression (LR)	76.8%	0.85
Optimizable Naive Bayes (NB)	74.2%	0.83
**Optimizable Support vector machine (SVM)**	**78.1%**	**0.85**
Optimizable k-nearest neighbors (KNN)	76.2%	0.83
Optimizable Ensemble	76.8%	0.76

## Discussion

The present study introduces a MATLAB® algorithm that first automatically detects the T-wave end, and then subsequently estimates QT interval (corrected and uncorrected), T-peak to T-end (TPE, corrected and uncorrected), T-wave area, T-wave duration and R-peak to T-peak amplitude ratio in digital ECGs. The performance of the algorithm was evaluated against manual measurements by specialists (as Gold Standard—GS) and the estimated QT by MUSE™ system (QT_MU), in 466 LQT1 and LQT2 patients. The exceptional performance of the algorithm, as shown here with reduced false positives, holds significant implications for clinical practice and patient care. In addition, our novel approach makes use of morphological parameters of the T-wave as listed above to improve classification of prolonged QT and can immensely contribute to the clinical assessment and management of these patients.

Most modern ECG machines offer automated measurements of ECG intervals to users. However, implemented algorithms have some limitations. For instance, the QT-interval is determined based on an averaged complex over time, resulting in the loss of temporal fluctuations and the dynamic nature of the QT-interval concerning changes in heart rate (HR). Additionally, the lack of transparency regarding the specifics of these algorithms poses a challenge to their users. Nevertheless, despite this limitation, many cardiologists rely on and trust the QTc interval provided by ECG machines. Here we show, using the Bland–Altman plot that a systematic bias in the QT measurement occurs when relying on ECG machine output only, which could lead to misdiagnosis of patients with LQTS. The GE MUSE™ system, although widely used in clinical settings, relies on proprietary algorithms, and may suffer from limitations due to the nature of its design and the complexity of ECG signal analysis. Our algorithm's unbiased and stable performance signifies its potential to surpass existing commercial solutions and set a new benchmark for QT measurements as well as calculation of other ECG parameters.

Manual assessment of the QT interval is time consuming for a physician’s daily routine and, in consequence, is often relied on values provided by the ECG software. Therefore, in practice, only one lead and one ECG complex often are selected for measurement. This is a questionable practice as one lead might not be representative. Indeed, results from this study clearly show a strong variability across channels, in terms of T-wave end detection. In general, automatic algorithms are objective, observer independent, and time sparing methods to overcome issues of manual measurements. Unfortunately, current implementations are either unavailable to the public or are not accurate enough. For example, [6] developed an algorithm to evaluate the QT interval based on the automated tangent method that can be applied beat-by-beat similarly to our algorithm. The authors found a small bias of −1.88 ms to + 3.39 ms when compared to manual measurement. This relatively small bias should be considered with caution however, as the authors included only very clear beats for the validation of their algorithm, whereas in our novel algorithm and validation approach, also noisy data were included, as in real-world data.

By including all possible ECG signals, noisy and non-noisy, we have detected that there are very few times in which the algorithm does not provide any estimate of the QT signal, whereas the MUSE (TM) system and the manual assessment was able to produce an estimate. Note that in these situations estimations are based on single ECG complex which are not stable.

The importance of correct ECG assessment in LQTS has been demonstrated in studies that aimed to detect LQTS patients with concealed phenotype, which means that patients have a genetic mutation but present with otherwise normal QT interval. In a study by Bos et al., deep neural networks of 12-lead ECG were used to distinguish patients with LQTS from those who were evaluated for LQTS but discharged as healthy (Bos et al., 2021). The authors found that using QTc alone, AUC was 0.82, whereas using all 12-leads, 10 s each, as an input to a deep neural network algorithm led to an AUC of 0.9, suggesting that additional parameters in the ECG signal could help to better identify LQTS patients. This result has been replicated [4] who showed using a novel convolutional neural network model resulting in a high accuracy rate (91.8%) in identification of concealed LQTS. Giudicessi and colleagues (2021) demonstrated that a deep neural network (DNN) is able to predict QTc using a database of over 1.6 Million 12-lead ECGs from 538,200 patients. A mean difference of –1.76 ± 23.14 ms was observed between 12-lead ECG–derived and 2-lead DNN–predicted QTc values, which seemingly provides high accuracy for QT estimation. The robust diagnostic performance in detecting Long QT Syndrome suggests that automated methods can be instrumental in clinical settings, especially for continuous monitoring using wearable devices. In another study, [2] used neural networks on ECG data to show that genotype positive LQTS patients can be accurately identified based on the ECG data only. Note that unlike our study, in which we take specific ECG parameters and investigate their influence on correct prolonged QT detection, the use of neural networks does not allow to deduce which parameters exactly influenced the correct estimation of the QT interval. Thus, conclusions regarding the specific mechanisms associated with ECG parameters are very limited.

The usage of T-wave morphology parameters has enhanced the ability of our algorithm to correctly detect prolonged QTc. Previous study by Immanuel et al. [7] showed that T-wave morphology based parameters enabled 90% discrimination between control and LQTS patients, compared to only 71% when the groups were classified based on QTc alone. The T-wave area reflects the total repolarization activity of the ventricles. In LQTS, repolarization is often abnormal, not just prolonged. Abnormal T-wave morphology, including a reduced or notched T-wave area, is a common finding in LQTS and can help differentiate subtypes [17]. The T-peak to T-end duration is a measure of transmural dispersion of repolarization—how different myocardial layers repolarize at different times. Increased TPE is associated with higher arrhythmic risk and is often prolonged in LQTS [5]. T-peak to R-peak amplitude ratio is a surrogate for T-wave amplitude abnormality relative to the main depolarization (R-wave). LQTS can produce low-amplitude or notched T-waves, making this ratio lower than in healthy controls. It can help distinguish LQTS from other causes of QT prolongation with normal T-wave amplitude [7]. A high ratio between TPE duration and QTc Interval indicates disproportionately increased dispersion of repolarization, which is highly arrhythmogenic and characteristic of LQTS [13]. This index helps distinguish between “global” (QTc prolongation) and “regional” (dispersion, TPE) repolarization abnormalities.

Here we observe that T-wave morphology parameters provide additional information beyond what the QT interval offers. Abnormal T-wave morphology (e.g., low amplitude, notching, biphasic shape) is common in LQTS—even when the QT interval isn’t overtly prolonged. Certain T-wave patterns (e.g., prolonged TPE, low T/R ratio) are associated with higher arrhythmia risk, even if the QTc interval is only mildly prolonged. Adding these markers allows better prediction of clinical outcomes, not just diagnosis.

### Clinical Implications and Limitations

Congenital LQTS is a life-threatening condition that requires early detection and intervention to prevent sudden cardiac events. Our algorithm offers a potential tool for highly accurate early identification of patients at risk, allowing for timely clinical intervention and improved patient outcomes.

At first glance, a major limitation of our algorithm may appear to be its comparatively low sensitivity (62%) relative to established systems such as MUSE™ system (90%). However, this lower sensitivity does not reflect a failure of the algorithm, but rather highlights a fundamental distinction between genotype-based classification and phenotypic QTc assessment. All patients in our LQTS cohort were genetically confirmed, yet a relevant subset, particularly among LQT1 and LQT2, displayed QTc intervals within normal limits at rest. These so-called “concealed LQTS” cases represent biologically silent phenotypes that, while genotype-positive, do not exhibit QTc prolongation at the time of recording.

Since our algorithm is designed to detect prolonged QTc, not the presence of an LQTS-causing mutation, these cases might be appropriately classified as “normal” from a repolarization perspective. As a result, when sensitivity is calculated against genotype as the ground truth, it underestimates the true performance of the algorithm in detecting electrocardiographic risk phenotypes.

A more clinically meaningful reference is the manually measured QTc interval, or Gold Standard (GS), which remains the most accurate method in expert hands. In our analysis, the MUSE™ system substantially overestimated QTc values and produced a very low specificity (59% vs. 89%). This in turn leads to more false positives and potentially unnecessary follow-ups and/or treatments. By contrast, our algorithm applies stricter signal quality criteria and integrates morphology-based T-wave logic, reducing the risk of false alarms.

Importantly, QTc interval duration and morphology remain central to clinical risk stratification, even among genetically confirmed LQTS patients. Recent work by Rieder et al. [14] has shown that both resting and post-exercise QTc duration, as well as markers of repolarization heterogeneity, correlate with symptom burden and arrhythmic risk. This underscores the importance of precise QTc measurement as a phenotype-driven biomarker, especially when genetic information alone is insufficient to guide management.

In light of this, our algorithm provides added value where manual QT measurement is difficult or inconsistent, such as in low-amplitude or biphasic T-waves, baseline drift, or ambiguous terminal repolarization. Its reproducibility and transparency make it a robust decision-support tool for clinicians, particularly in high-throughput or resource-limited environments.

In summary, the lower genotype-based sensitivity of our algorithm reflects biological heterogeneity rather than technical insufficiency. Its specificity, reproducibility, and clinically aligned design, along with its ability to operate transparently and modularly, position it as a useful adjunct in modern QTc analysis workflows. Rather than replacing clinical judgment, it offers a phenotype-aware, expert-guided bridge between manual gold standard assessment and automated ECG processing.

### Future Directions

While the proposed algorithm has demonstrated significant advancements in T-wave end detection, there are several avenues for further improvement and exploration. Increasing the dataset size and diversity could enhance the algorithm's robustness and generalizability across various patient populations and ECG acquisition settings.

The high accuracy of QT interval measurement is of utmost importance for diagnosis and risk assessment in cardiac patients not only for congenital LQTS but also for evaluating acquired LQTS, such as risk for life-threatening drug-induced torsade de pointes arrhythmias. The algorithm's accuracy can lead to more targeted interventions, minimizing unnecessary or life-threatening treatments and interventions, thereby optimizing patient care and potentially reducing healthcare costs.

## Conclusion

In conclusion, the presented MATLAB® algorithm for automatic T-wave end detection and estimation of QT interval as well as other relevant parameters in digital ECGs exhibit high accuracy. Its reduced false positives and the ability to detect prolonged QT underscores its clinical significance in improving patient care and decision-making. This algorithm opens-up new possibilities for enhancing cardiac diagnostics and monitoring, and it holds great promise for future advancements in combined genetic and ECG analysis.

## Supplementary Information

Below is the link to the electronic supplementary material.

Supplementary file1 (DOCX 1.20 MB)

## Data Availability

All data and code-source produced in the present study are available upon reasonable request to the corresponding author.

## Footnotes

1 https://github.com/DFNOsorio/GEMuseXMLReader..

2 https://www.researchgate.net/publication/313673153_Matlab_Implementation_of_Pan_Tompkins_ECG_QRS_detector
