# Supplementary material for: A MATLAB Algorithm to Automatically Estimate the QT Interval and Other ECG Parameters and Validation Using a Machine Learning Approach in Congenital Long-QT Syndrome
Source: J Cardiovasc Transl Res. 2025 Sep 30;18(5):1470–81. doi: 10.1007/s12265-025-10693-0 (PMC12630201; doi:10.1007/s12265-025-10693-0)

**Supplementary Materials - Part1**

In the following we present 5 cases in which the ECG was too noisy for the algorithm to detect any T-wave. We present also the estimated GS and the QT measured by the MUSE algorithm. All values are uncorrected.

Subj1 (UKM0003) LQT2, QT_GS = 500ms, QT_MU = 516ms


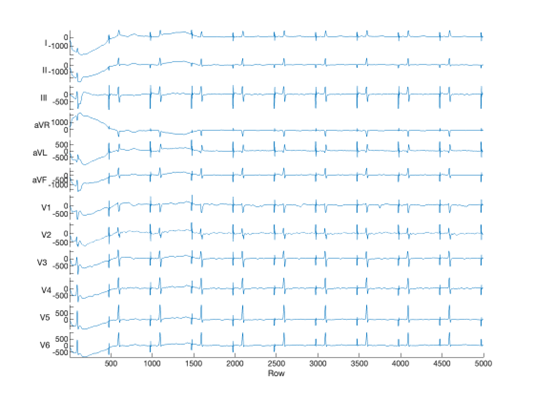


Subj2 (UKM0048) LQT2, QT_GS = 524ms, QT_MU = 524ms
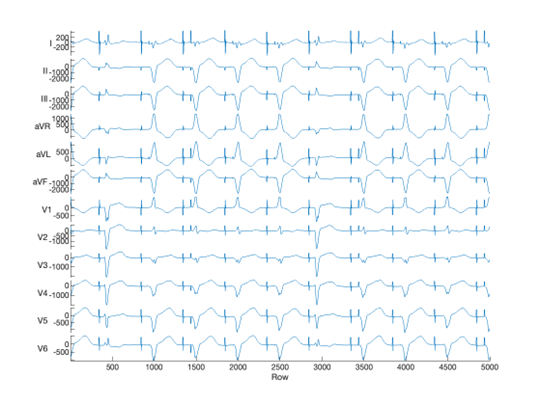


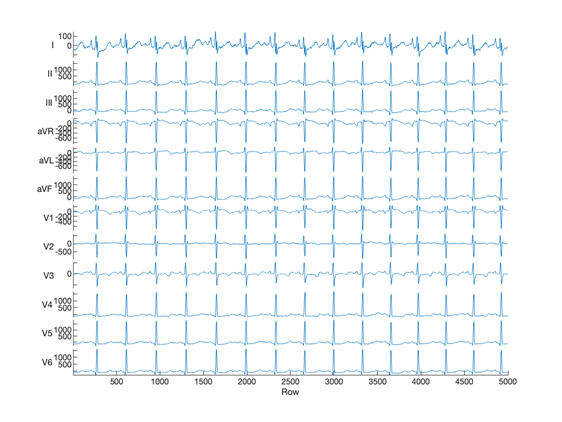


Sub3 (UKM0232) LQT1, QT_GS = 410ms, QT_MU = 414ms


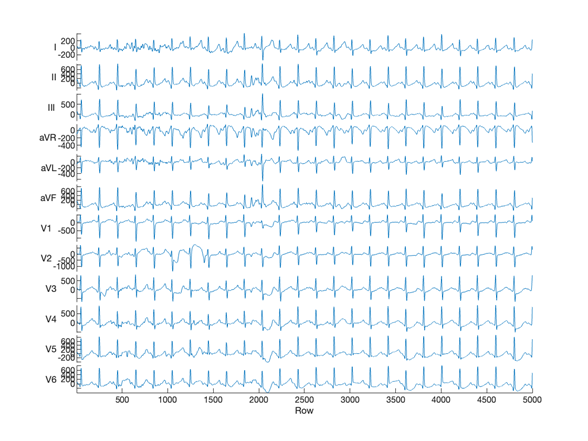


Subj4 (UKM0267) LQT1, QT_GS = 280ms, QT_MU = NaN


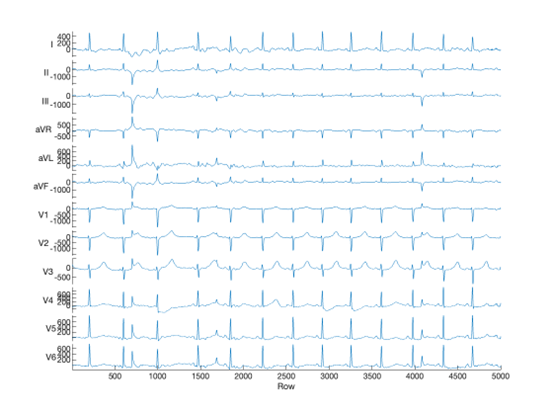


Subj5 (UKM0299) LQT1, QT_GS = 440ms, QT_MU = 432ms

Summary

Clearly the signals presented above are too noisy to detect any T-wave. In some cases, there has been a single beat where detection was possible and this was the source of the value measured by the experts and by the MUSE algorithm.

Supplementary Materials - Part2

Subj1 (UKM0191) LQT2, channel: V2

Su
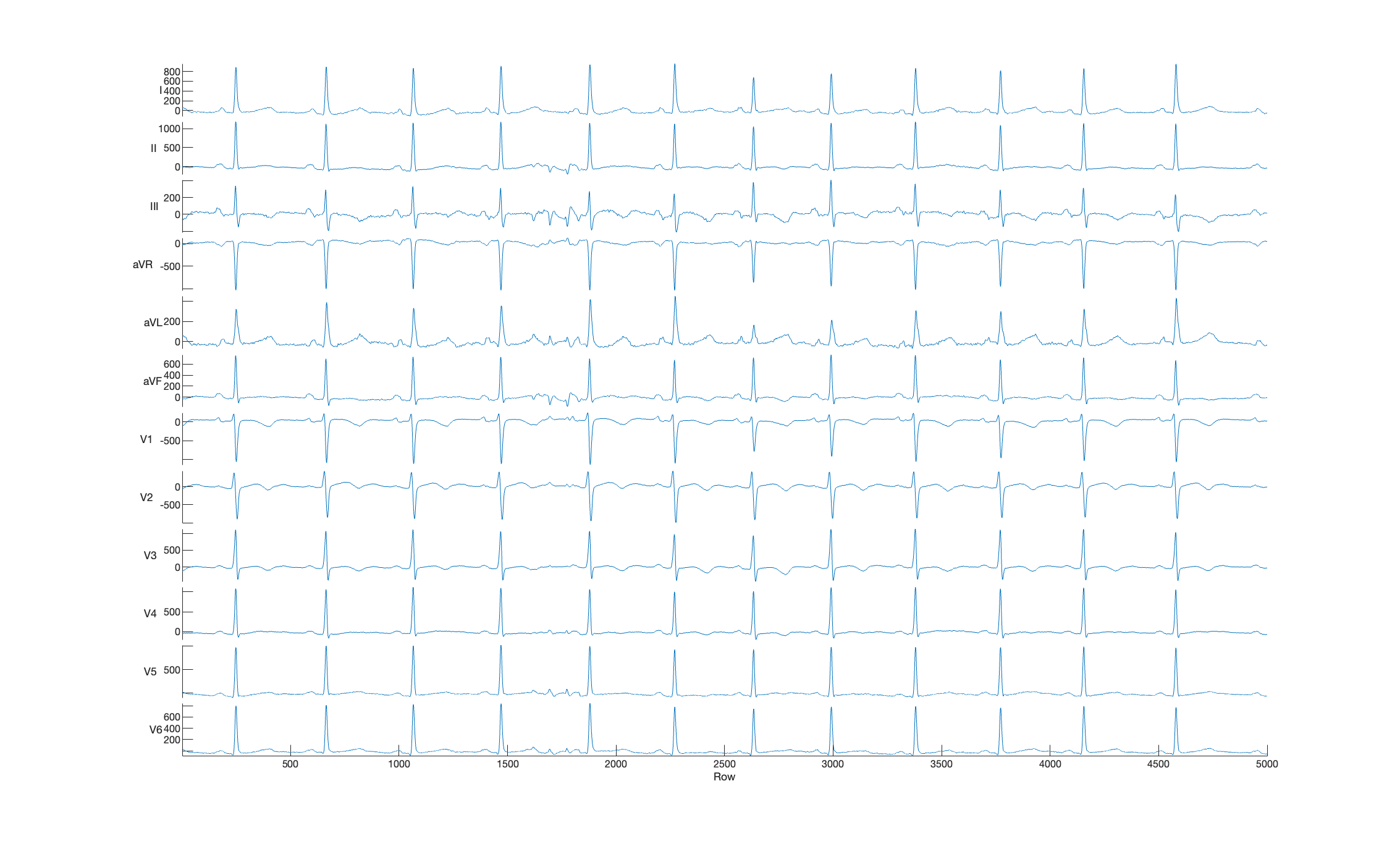
bj2 (UKM0195) LQT2, channel: II


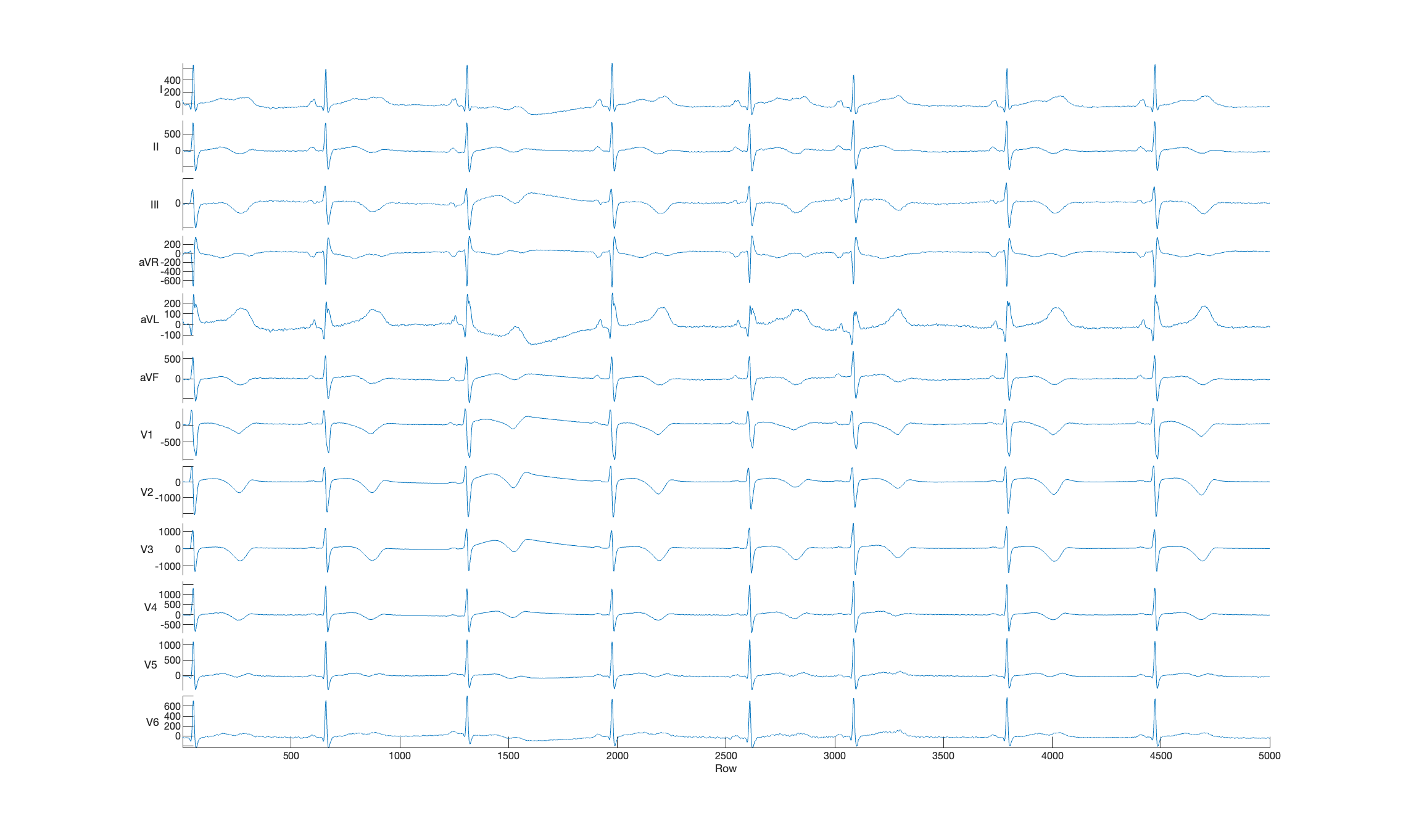


Subj3 (UKM0243) LQT1, channel: V2

Subj4 (UKM0410) LQT1, channel: V2
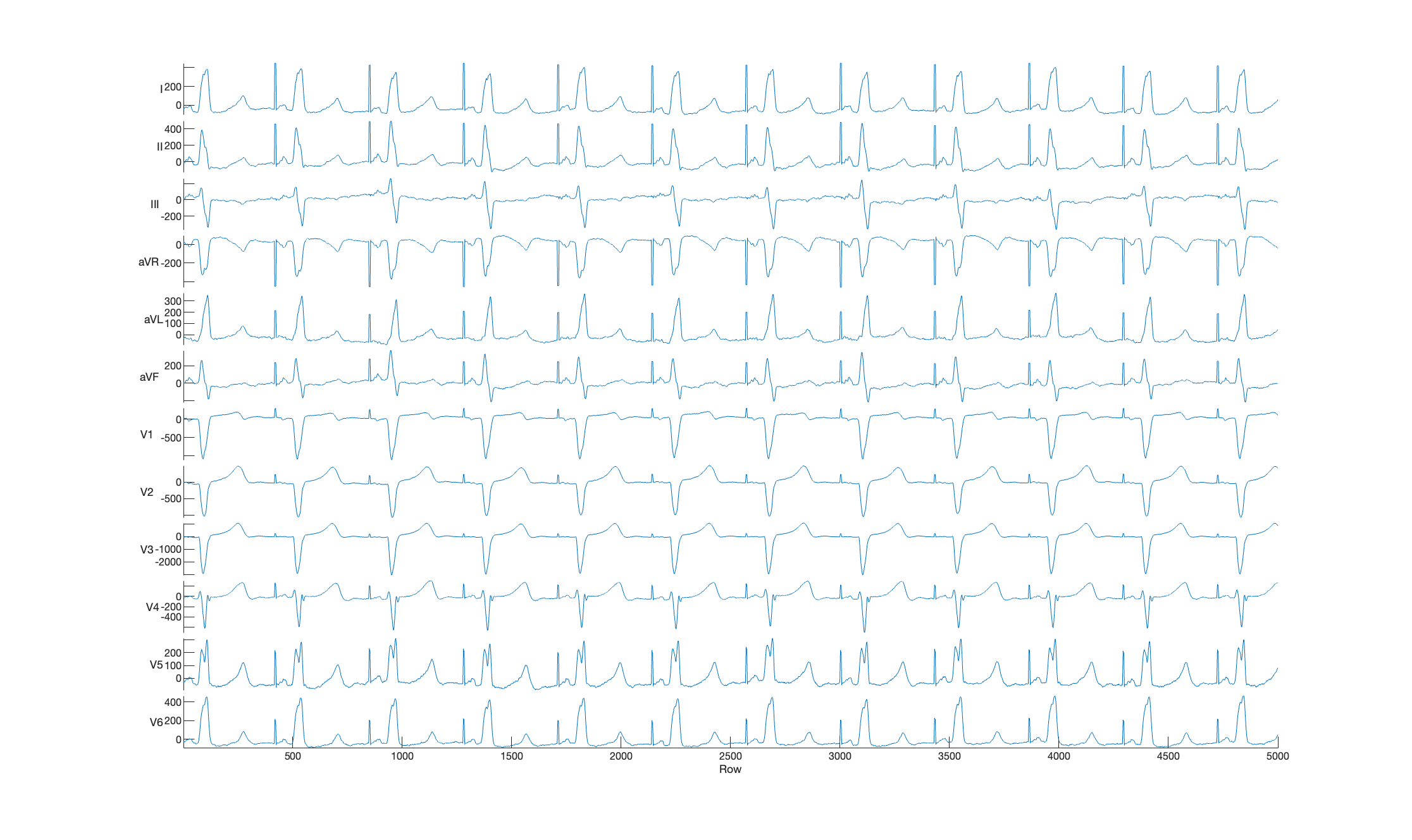


Subj5 (UKM0550
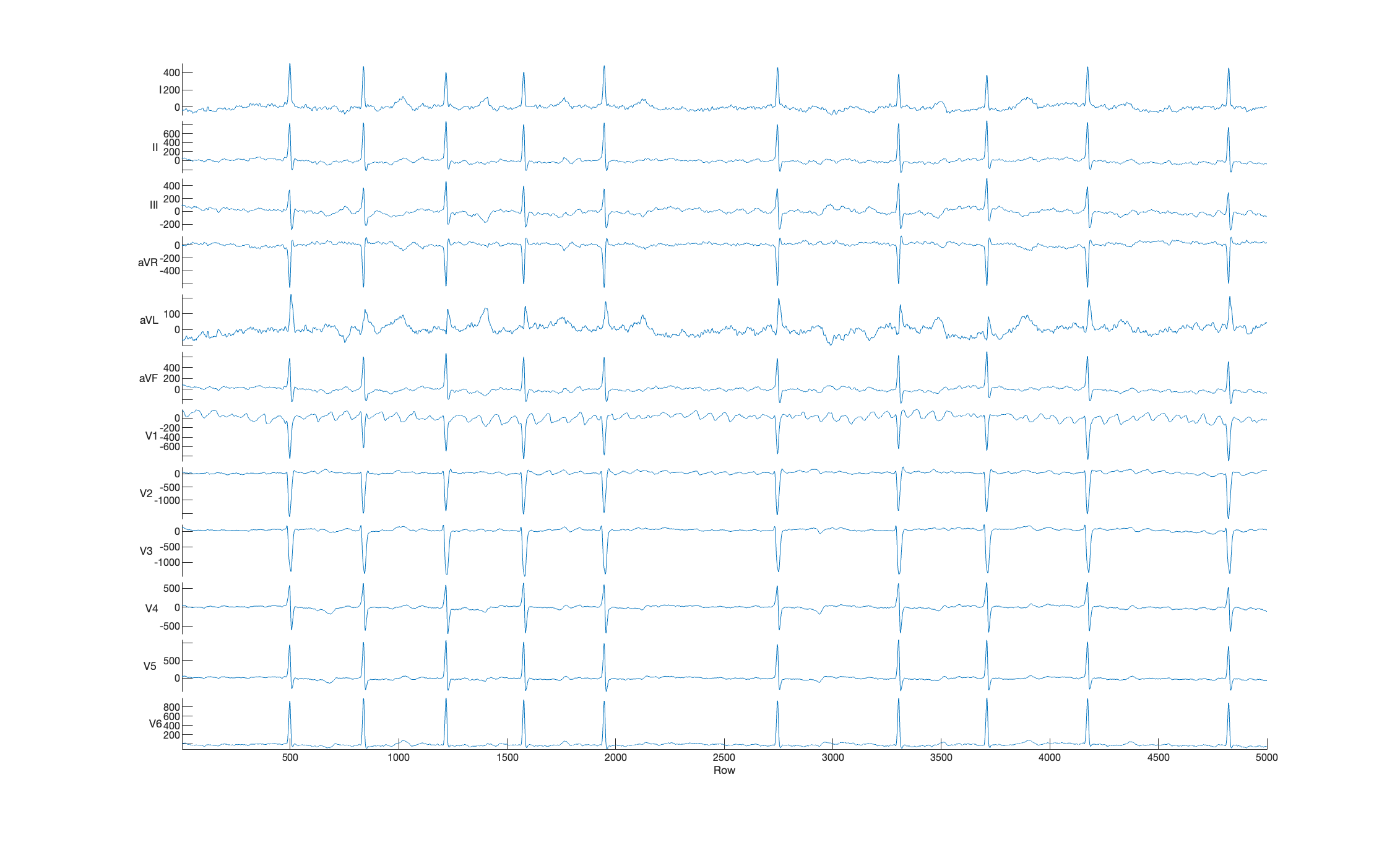
) LQT2, channel: II


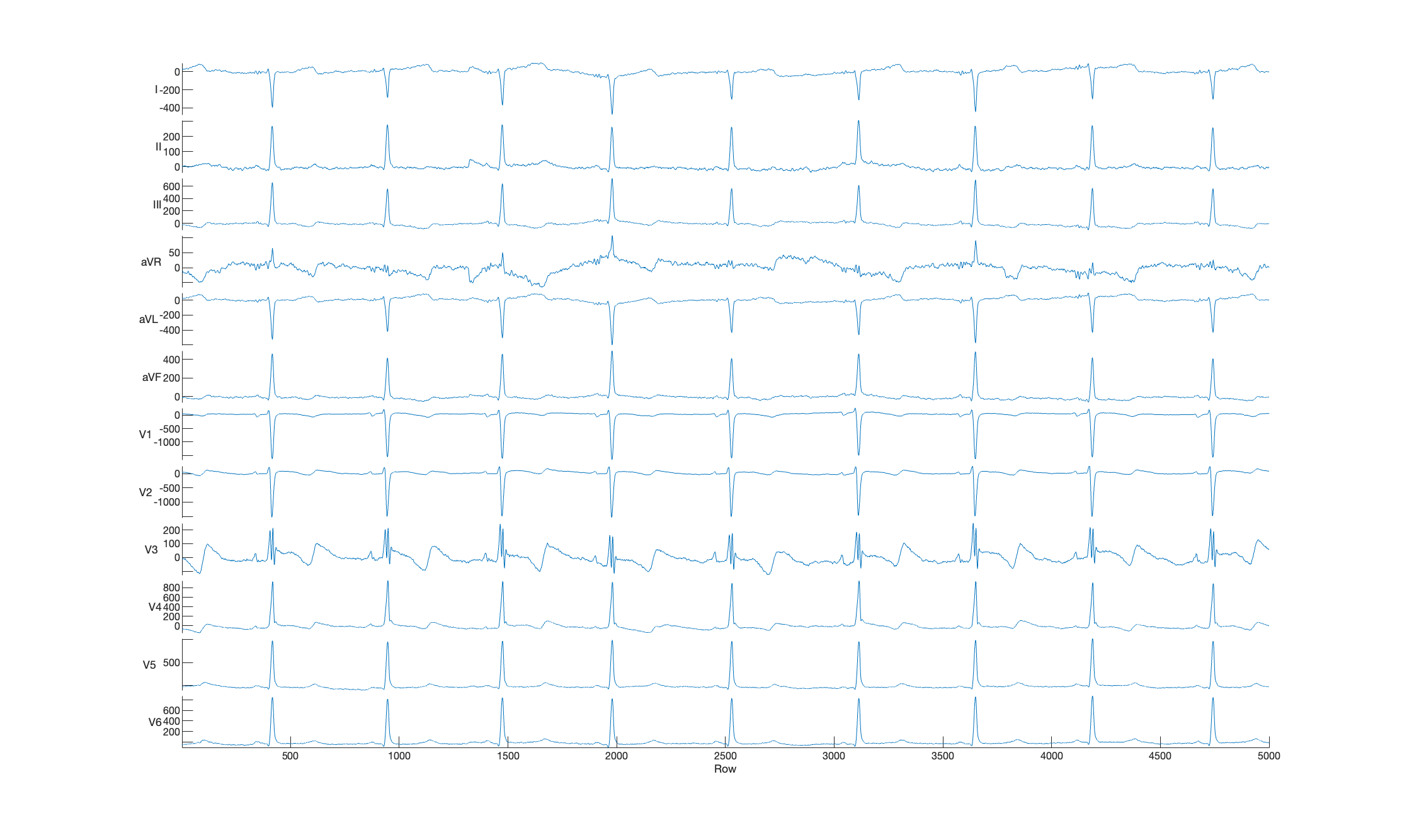


Subj6 (UKM0597) LQT2, channel: V2

Subj7 (UKM0641)
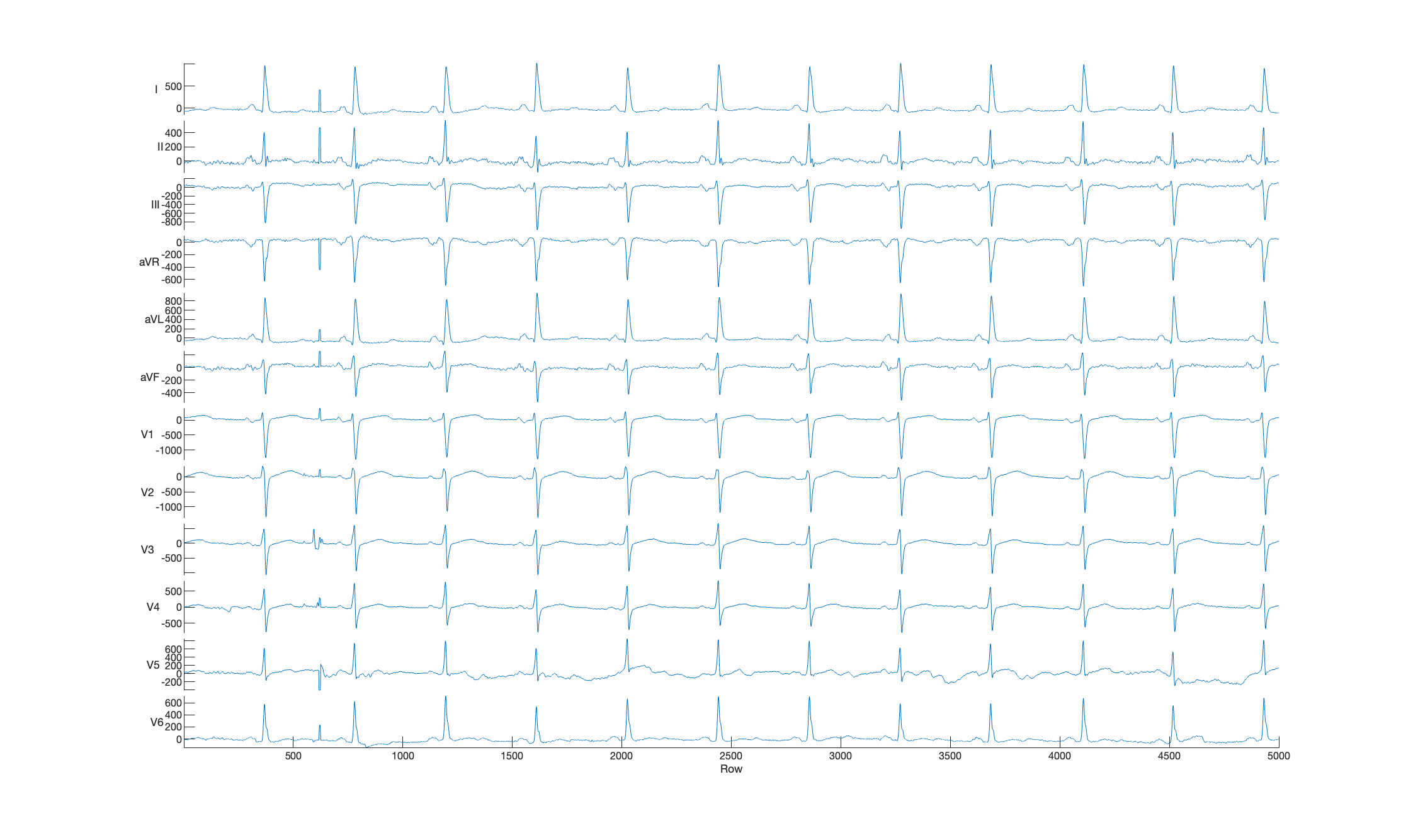
 LQT2, channel: V5


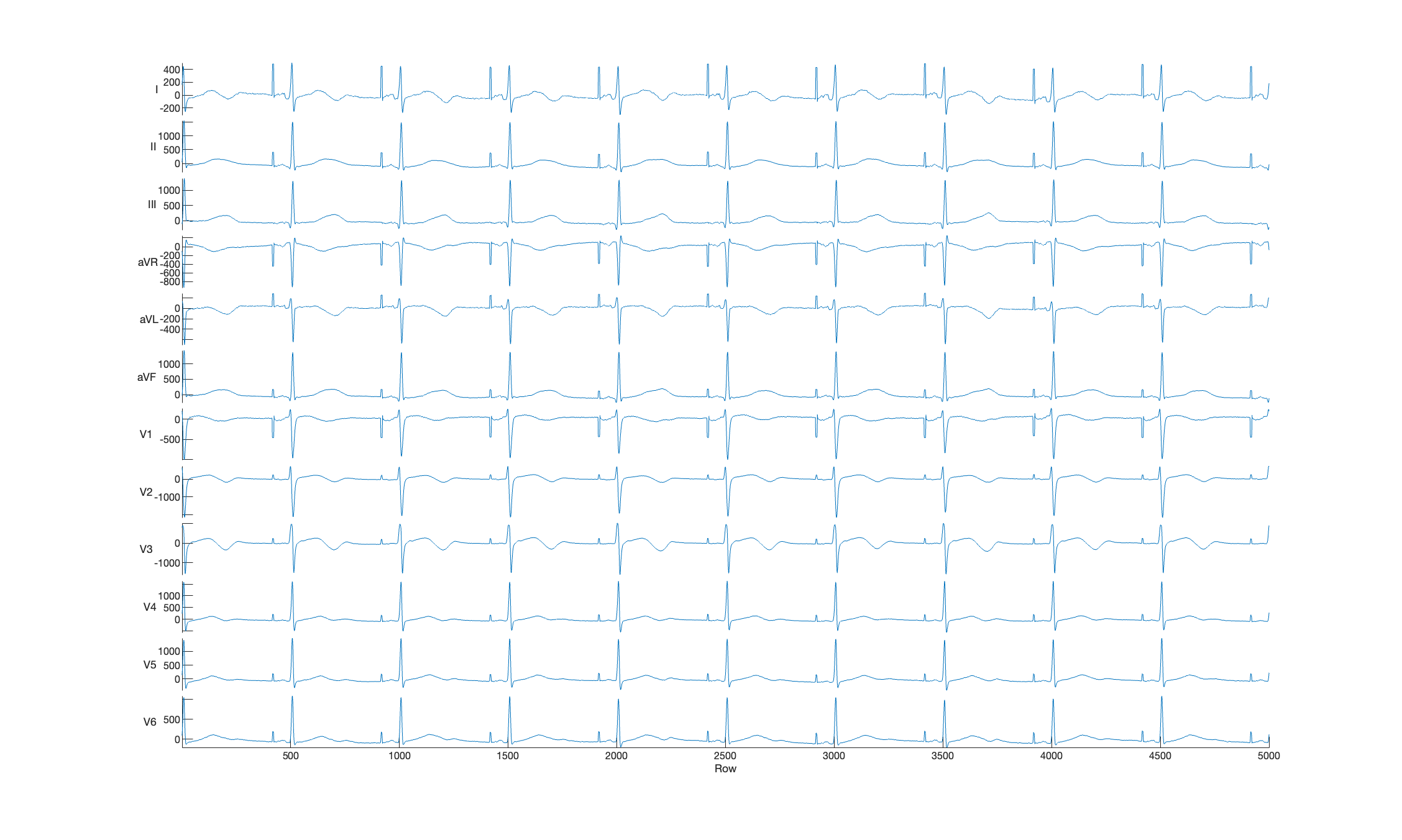

Supplement: Supplementary file 1 — Supplementary file1 (DOCX 1.20 MB) [file 12265_2025_10693_MOESM1_ESM.docx]
